# Identification of Tuberculosis Susceptibility Genes with Human Macrophage Gene Expression Profiles

**DOI:** 10.1371/journal.ppat.1000229

**Published:** 2008-12-05

**Authors:** Nguyen Thuy Thuong Thuong, Sarah J. Dunstan, Tran Thi Hong Chau, Vesteinn Thorsson, Cameron P. Simmons, Nguyen Than Ha Quyen, Guy E. Thwaites, Nguyen Thi Ngoc Lan, Martin Hibberd, Yik Y. Teo, Mark Seielstad, Alan Aderem, Jeremy J. Farrar, Thomas R. Hawn

**Affiliations:** 1 Oxford University Clinical Research Unit, Hospital for Tropical Diseases, Ho Chi Minh City, Vietnam; 2 Hospital for Tropical Diseases, Ho Chi Minh City, Vietnam; 3 Centre for Tropical Medicine, Nuffield Department of Clinical Medicine, Oxford University, Oxford, United Kingdom; 4 Institute for Systems Biology, Seattle, Washington, United States of America; 5 Centre for Molecular Microbiology and Infection, Imperial College, London, United Kingdom; 6 Pham Ngoc Thach Hospital for Tuberculosis and Lung Disease, Ho Chi Minh City, Vietnam; 7 Genome Institute of Singapore, Agency for Science, Technology, and Research, Singapore; 8 Wellcome Trust Centre for Human Genetics, Oxford University, Oxford, United Kingdom; 9 University of Washington School of Medicine, Seattle, Washington, United States of America; University of California San Francisco, United States of America

## Abstract

Although host genetics influences susceptibility to tuberculosis (TB), few genes determining disease outcome have been identified. We hypothesized that macrophages from individuals with different clinical manifestations of *Mycobacterium tuberculosis* (*Mtb*) infection would have distinct gene expression profiles and that polymorphisms in these genes may also be associated with susceptibility to TB. We measured gene expression levels of >38,500 genes from *ex vivo Mtb*-stimulated macrophages in 12 subjects with 3 clinical phenotypes: latent, pulmonary, and meningeal TB (n = 4 per group). After identifying differentially expressed genes, we confirmed these results in 34 additional subjects by real-time PCR. We also used a case-control study design to examine whether polymorphisms in differentially regulated genes were associated with susceptibility to these different clinical forms of TB. We compared gene expression profiles in *Mtb*-stimulated and unstimulated macrophages and identified 1,608 and 199 genes that were differentially expressed by >2- and >5-fold, respectively. In an independent sample set of 34 individuals and a subset of highly regulated genes, 90% of the microarray results were confirmed by RT-PCR, including expression levels of CCL1, which distinguished the 3 clinical groups. Furthermore, 6 single nucleotide polymorphisms (SNPs) in CCL1 were found to be associated with TB in a case-control genetic association study with 273 TB cases and 188 controls. To our knowledge, this is the first identification of CCL1 as a gene involved in host susceptibility to TB and the first study to combine microarray and DNA polymorphism studies to identify genes associated with TB susceptibility. These results suggest that genome-wide studies can provide an unbiased method to identify critical macrophage response genes that are associated with different clinical outcomes and that variation in innate immune response genes regulate susceptibility to TB.

## Author Summary

Although TB is a leading cause of death worldwide, the vast majority of infected individuals are asymptomatic and contains the bacillus in a latent form. Among those with active disease, 80% have localized pulmonary disease and 20% have disseminated forms. TB meningitis (TBM) is the most severe form of TB with 20–25% of sufferers dying, and of the survivors, many have disability. We currently do not understand the host factors that regulate this diverse spectrum of clinical outcomes. We hypothesized that variation in innate immune gene function is an important regulator of TB clinical outcomes. We measured the mRNA expression levels of >38,500 genes in macrophages taken from people with a history of latent, pulmonary, or meningeal TB and found genes with unique activation patterns among the clinical groups. Furthermore, we studied one of these genes further and found that CCL1 polymorphisms were associated with pulmonary TB (PTB) but not other types of TB disease. To our knowledge, this is the first study to combine mRNA expression studies with genetic studies to discover a novel gene that is associated with different clinical outcomes in TB. We speculate that this approach can be used to discover novel strategies for modulating immune function to prevent adverse outcomes in TB.

## Introduction

TB, a leading cause of death worldwide, is characterized by different clinical forms including latent TB (LTB), localized pulmonary infection, and various forms of extrapulmonary TB including TBM. 90% of people infected with *Mtb* have latent infection with no symptoms and an immune response that contains the bacilli. In 10% of infected individuals, symptoms develop and most commonly manifest as pulmonary disease, which accounts for 80% of all forms of TB disease [1]. TBM develops in around 1% of all cases of active TB [1] and is the most severe form with mortality rates of 20–25% and high rates of neurological sequelae in many of those who survive [2],[3]. Although only 10% of individuals who are infected with *Mtb* develop active disease, it is not known which immune responses are associated with susceptibility or resistance. In addition, it is not known why some individuals have disseminated TB that spreads to the meninges and central nervous system, while most people have localized disease in the lungs. Although environmental exposures, pathogen virulence traits, and host genetics have the potential to influence the different clinical manifestations of TB, it is not currently understood which factors are the most important [4].

Several lines of evidence, including twin and genome-wide linkage studies, suggest that host genetics strongly influences susceptibility to TB [5]–[9]. Candidate gene association studies have implicated common polymorphisms in genes that may influence the development of TB [10],[11]. Although there is potential for candidate gene study designs to be successful when sample sizes are sufficient and case and control groups are accurately defined, candidate genes are usually selected from lists of genes with known functions. A fundamental problem with this strategy is an inherent selection bias dominated by well-characterized genes. Furthermore, many genes are selected based on phenotypes identified from *in vivo* murine studies. Although mouse studies have provided powerful methods to dissect TB immunopathogenesis, the murine system models primary, progressive disease, which is only one of several phenotypes observed in humans. There are no well-established murine models of latent infection or the various types of disseminated disease, including TBM. *Mtb* intrathecal infection of rabbits recapitulates some of the inflammatory pathology but does not provide insight into the steps in immunopathogenesis involved in dissemination and invasion of the central nervous system [12]. To identify genes involved in TBM pathogenesis and to avoid gene selection bias, we chose to directly examine humans with different clinical types of TB with an array-based method to identify candidate genes.

Macrophages mediate the host innate immune response to *Mtb* through pathogen recognition and activation of an inflammatory response. *Mtb* resides in the macrophage phagolysosome, where it evades the immune response in the majority of infected individuals. Successful containment of *Mtb* replication results in LTB with no clinical symptoms, which depends on stimulation of innate and adaptive immune responses that lead to macrophage activation, formation of granulomas and elimination of the bacilli. In contrast, failure to contain bacilli replication is associated with active pulmonary disease and/or the development of disseminated disease. We hypothesized that different macrophage responses to *Mtb* are associated with distinct clinical outcomes that are genetically regulated.

Expression microarrays have been previously used to examine gene expression profiles in the immune response to TB [13]–[17]. None of these studies attempted to distinguish different clinical forms of active TB such as pulmonary and meningeal disease. In addition, the sample sizes were generally small and the findings were often not validated in independent sample sets. Finally, these previous approaches were not coupled with human genetic studies to examine the clinical significance associated with variation in the identified genes. In this manuscript, we examined *ex vivo Mtb*-stimulated monocyte-derived macrophages (MDMs) from subjects with pulmonary, meningeal and latent infection. We attempted to find unique gene expression profiles to determine whether clinical phenotypes in TB are associated with distinct early macrophage responses to *Mtb* stimulation. We then used a case-control genetic association study to examine whether genetic variation of these selected genes was associated with susceptibility to *Mtb*.

## Materials and Methods

### Human subjects

TBM subjects were recruited as part of a larger clinical study at the Hospital for Tropical Diseases, in Ho Chi Minh City (HCMC), Vietnam [18]. All subjects were >14 years of age and HIV-negative. TBM patients were described as having clinical meningitis (defined as nuchal rigidity and abnormal cerebrospinal fluid parameters) in addition to having a positive Ziehl-Neelsen stain for acid-fast bacilli and/or *Mtb* cultured from the cerebrospinal fluid. Subjects were treated for TBM and were clinically well (recovered for >3 years) when samples for this study were taken. For PTB subjects, samples were taken from individuals who had been previously treated and had recovered from uncomplicated PTB (no evidence of miliary or extrapulmonary TB). LTB subjects were defined as highly exposed individuals who had no history of active TB disease. LTB subjects were healthy nursing staff members who had worked at Pham Ngoc Thach Hospital for Tuberculosis and Lung Disease, HCMC, Vietnam for more than 20 years. They were tested for *Mtb* exposure using an ESAT-6 and CFP-10- specific IFN-γ ELISPOT assay using a previously described method [19].

For the initial microarray study, twelve subjects were enrolled with three clinical forms of TB; TBM (n = 4), PTB (n = 4) and LTB (n = 4). All of the LTB subjects tested positive in the ESAT-6 and/or CFP-10- specific IFN-γ ELISPOT assay, suggesting previous or current infection with *Mtb*. An extended sample set containing 34 subjects with TBM (n = 10), PTB (n = 12) and LTB (n = 12) was used in validation experiments. Of the 12 LTB subjects, 10 were IFN-γ ELISPOT positive according to our defined cut-off [at least 10 spot forming units (SFU) more than the negative PBS control and at least twice as many SFU as the negative PBS control]. The 2 IFN-γ ELISPOT indeterminate LTB subjects had borderline responses (6.7 SFU with a ratio of 2 and 6 SFU with a ratio of 2.5) which were considerably higher than an unexposed population (average of −2.8 SFU with a ratio of 0.8).

For the case-control genetic association study the cohort of TBM (N = 114) and PTB (N = 159) patients, and population controls (cord blood; N = 188) has been previously described [20].

All samples came from unrelated individuals who were ethnic Vietnamese Kinh, as assessed by questionnaire. Written informed consent was obtained from each patient. Protocols were approved by human subjects review committees at the Hospital for Tropical Diseases and Pham Ngoc Thach Hospital for Tuberculosis and Lung Disease, Ho Chi Minh City, Vietnam. Ethical approval was also granted by the Oxford Tropical Research Ethics Committee, UK (OXTREC), The University of Washington Human Subjects Committee (USA) and the Western Institutional Review Board (USA).

### *Ex vivo* generation and stimulation of MDMs

Peripheral blood mononuclear cells (PBMCs) were separated from heparinized whole blood by Lymphoprep (Asix-Shield, Norway) gradient centrifugation according to the manufacturer's protocol. From 20 ml of blood we obtained approximately 1–1.5×10^7^ PBMCs. To derive monocytes, PBMCs were plated in Nunclon Suface 6-well plates (Nunc, Denmark) containing RPMI-1640 (Sigma, Germany) with 10% heat-inactivated fetal calf serum (FCS; Sigma, Germany), 2 mM L-glutamine and 100 units of penicillin for 2 hours at 37°C. Non-adhered cells were removed by washing with phosphate buffered saline (PBS) containing 3% FCS and adhered cells were incubated for 5 days at 37°C, 5% CO_2_ to obtain MDMs. Cells were subsequently stimulated with PBS or 5 µg/ml of an irradiated, soluble, whole cell lysate of *Mtb H37Rv* [obtained from the Mycobacteria Research laboratories at Colorado State University, USA (http://www.cvmbs.colostate.edu/microbiology/tb/top.htm)] for 4 hours before RNA extraction. Pilot studies indicated that 5 µg/ml was an optimal dose for stimulating TNF-α production.

### RNA preparation and microarray hybridization

RNA was extracted from macrophages using Trizol according to the manufacturer's protocol (Invitrogen, USA), dissolved in RNase-free water and stored at −70°C until use. Total RNA (100 ng) was reverse transcribed to cDNA, amplified, labeled, and hybridized to the Human Genome U133 Plus 2.0 Array (Affymetrix, USA), according to the manufacturer's instructions. This array contains probe sets to measure the expression level of 47,000 transcripts, including 38,500 well-characterized human genes. Twelve *Mtb*-stimulated (TBM n = 4, PTB n = 4, and LTB n = 4) and 12 PBS-stimulated (hereafter called unstimulated) samples were hybridized to the array. The microarray data is publicly available at ArrayExpress, EMBL-EBI (Submission in progress, awaiting Accession number; http://www.ebi.ac.uk/microarray-as/aer/?#ae-main0).

### Microarray data processing and statistics

After normalization of the expression values, the data from 12 *Mtb*-stimulated samples were compared with the 12 unstimulated samples. Data were considered significant when (1) the false discovery rate (FDR) from the Significance Analysis of Microarray (SAM) analysis for the comparison of stimulated and unstimulated expression values was <0.05, and (2) the *P* value of the comparison between stimulated versus unstimulated expression values by Student's t-test was <0.05. In order to focus on highly regulated genes, we also restricted the majority of the analysis to genes with changes in expression levels of at least 2-fold. To compare gene expression levels among the three different clinical types of TB, we first calculated the fold stimulation of each gene for each individual by dividing the *Mtb*–stimulated value by the unstimulated control values. The averages of the 4 samples in each clinical group were calculated and then compared to the other groups by calculating the ratios of expression levels. The pair-wise comparisons included TBM vs. PTB, TBM vs. LTB, and PTB vs. LTB. SAM [20] was used to derive the FDR for microarray data, which is the proportion of genes likely to have been identified as significant by chance. Student's t-test and analysis of variance (ANOVA) were used to compare mean expression levels. To analyze expression patterns in multiple genes simultaneously we used Hierarchical Clustering [21]. Analyses were performed using MultiExperiment Viewer (MeV version 4.0, USA) [22] and SPSS (version 14.0, USA).

### Real-time quantitative PCR

Taqman real time PCR was used to validate microarray gene expression results. cDNA was synthesized from total RNA samples using reverse transcription with Superscript II following the manufacturer's protocol (Invitrogen, USA). A commercial Low Density Array (LDA) format with Taqman probes and primers was then used for PCR validation (Applied Biosystems, USA). Expression levels in 88 genes [86 selected genes and 2 controls (GAPDH; Hs00237184_m1 and Hs00266705_g1)] were examined in each sample according to the manufacturer's instructions. CCL1 gene expressions on human and mice were examined by using Taqman probes and primers (Applied Biosystems, USA). Samples were normalized to GAPDH and analyzed by using either Applied Biosystems SDS 2.1 Relative Quantification software or an Excel spreadsheet to perform relative quantification analysis.

### CCL1 chemokine assay

PBMC were isolated from whole blood and cytokine assays were prepared by plating 10^5^ cell per well with RPMI (Life Technologies) in a 96-well dish, stimulating for 24 hours, and then harvesting supernatants. Stimuli included: Ultrapure lipopolysacharide (LPS) at 100 ng/ml, from *Salmonella minnesota* R595 (List Biological Labs, Inc.), *Mtb* H37Rv whole cell lysate, *Mtb* H37Rv cell wall fraction and *Mtb* H37Rv cytosol fraction (TB Vaccine Testing and Research Materials Program at Colorado State University). Chemokine levels were determined with a sandwich ELISA technique (Duoset, R&D Systems, Minneapolis, MN).

### Case-control genetic association study and statistics

SNPs in the CCL1 and CCR8 genes were genotyped in patients with TBM (N = 114), PTB (N = 159), and in Vietnamese Kinh population controls (N = 188). This genotyping was performed as part of a larger genome-wide genetic association study of TB using the Affymetrix 250K NspI Chip (unpublished). The whole genome SNP genotyping was performed according to the manufacturer's specifications and the data obtained was analyzed following rigorous quality control. Briefly, data quality control was performed using DM, BRLMM, RELPAIR, and manual viewing of cluster plots prior to statistical analysis. STRUCTURE and Eigentstrat were also used to analyse the population structure of the sample set. Genomic DNA quality was first assessed with 50 control SNPs and only samples with a call rate of greater than 93% were studied further. For each polymorphism in the full dataset, filter criteria were applied that included <5% missing values and HWE *P* value>10^−5^. Power for this study was calculated by using Power Calculator for Genetic Studies, CaTS version 0.0.2 (http://www.sph.umich.edu/csg/abecasis/CaTS). With a sample size of controls = 188 and PTB = 159 we have 82% power to detect an effect with an odds ratio of 2 for SNPs with an allele frequency of 10% and significance level of 0.01. With a sample size of controls = 188 and TBM = 114, we have a power of 71% to detect the same effects.

Genotyping was also carried out on selected CCL1 SNPs using a larger sample set TBM (N = 162), PTB (N = 175), and in Vietnamese Kinh population controls (N = 380). This was performed by a MassARRAY™ technique (Sequenom, San Diego, USA) using a chip-based matrix-assisted laser desorption/ionization time-of-flight mass spectrometer as previously described [18]. All of the CCL1 SNPs genotyped by Sequenom were in Hardy Weinberg Equilibrium (HWE) (*P*>0.05) in population controls.

Univariate analysis was performed for categorical variables with a Chi-Square test. Two-sided testing was used to evaluate statistical significance.

## Results

### Gene expression profiles in *Mtb*-stimulated and unstimulated MDMs

We hypothesized that macrophages from individuals with different TB clinical phenotypes have distinct gene expression profiles in response to *Mtb* stimulation. All subjects with pulmonary and meningeal disease had been treated and were free of symptoms at the time of venipuncture. Gene expression of MDMs from subjects with three clinical forms of TB including LTB, PTB, and TBM (n = 4 in each group) was examined by microarray. MDMs were stimulated either with a whole cell lysate of *Mtb H37Rv* or PBS for 4 hours. RNA expression was analyzed using a Human Genome U133 Plus 2.0 Array (Affymetrix, USA) which contains probe sets for 47,000 transcripts including 38,500 well-characterized human genes. We compared RNA transcription levels in *Mtb*-stimulated (n = 12) versus PBS-stimulated (n = 12) MDMs. 1,608 genes with a FDR of <5% and a *P* value of <0.05 by Students's t-test were differentially expressed by greater than 2-fold (Table 1). Of these genes, 1,260 were up-regulated and 348 genes were down-regulated. A list of the 1,608 genes that were differentially expressed in the two groups (n = 24) with their mean expression intensities, FDR and *P* values are presented in Table S1. 74 genes were up-regulated more than 10-fold, whereas only one gene was down-regulated by greater than 10-fold (Table 1). We used PANTHER (Protein Analysis Through Evolutionary Relationships; http://www.pantherdb.org/) to analyze the molecular functions and biological processes of genes induced and repressed in *Mtb*-stimulated MDMs. The changes in gene expression induced after stimulation contained 144 (8.4%) immunity and defense genes, including cytokines, chemokines, and receptors. Thirty six of these genes (25%) were up-regulated more than 10-fold. In contrast, no immunity and defense genes were repressed more than 10-fold. Other categories included; development (6.7%), protein and nucleic metabolism (19.2%) and signal transduction (11.9%). By comparison to the entire human genome, the proportion of immunity and defense genes is 5.2%. Percentages of other categories include: development (8.5%), protein and nucleic metabolism (25.1%) and signal transduction (13.4%).

**Table 1:** Gene expression ratios in *Mtb* stimulated MDMs.

	ratio>10	5<ratio<10	2<ratio<5
	(# genes)	(# genes)	(# genes)
**All TB** a			
Up regulated	74	111	1,075
Down regulated	1	13	334
Total	**75**	**124**	**1,409**
**TB clinical phenotypes** b			
TBM/PTB	6	27	450
PTB/TBM	4	14	500
TBM/LTB	5	55	1,763
LTB/TBM	2	35	1,474
PTB/LTB	8	46	1,688
LTB/PTB	8	51	1,519
Total	**33**	**228**	**7,394**

a All TB; the ratio indicates the mean of *Mtb* stimulated samples (n = 12) divided by the mean of PBS-stimulated samples (n = 12) when analyzed with the U133 Plus 2.0 Array.

b TB clinical phenotypes; six pairwise comparisons were derived between 2 clinical phenotypes of either TBM, PTB, or LTB. Ratios derived by first dividing the mean value of *Mtb* stimulated samples (n = 4) by the PBS-stimulated samples (n = 4) in each group and then calculating ratios of expression levels between two groups.

### Gene expression in different clinical phenotypes of TB (TBM, PTB, LTB)

To examine whether individuals with different clinical forms of TB have distinct gene expression profiles, we calculated the fold stimulation of each gene for each individual (dividing *Mtb* stimulated value by the unstimulated value) and then calculated the ratios of gene expression levels in each pair of TB forms. Six pair-wise comparisons in Table 1 show the change of gene expression between disease types (in fold change). 33 genes were differentially expressed between disease types with a ratio >10 and 228 genes had a ratio from 5 to 10.

In Table 2, half of the genes with a ratio >10 (16/33) were immunity genes including chemokines, cytokines and immune receptors. Others such as MMP1 and HAS1 are involved in degrading the extracellular matrix [23]. When all 3 clinical groups were compared, 16 genes had expression values that were significantly different (CXCL5, EREG, TNIP3, INHBA, HAS1, MGC10744, CCL1, KCNJ5, SERPINB7, HS3ST2, APOBEC3A, MYO10, SLC39A8, CXCL11, F3, and DUSP5, ANOVA <0.05). We then compared expression values of pairs of clinical groups. There were 11 genes highly expressed in TBM in comparison to other forms of TB (Table 2). 6/11 genes (IL1B, CXCL5, EREG, TNIP3, CCR2, and INHBA) were significantly induced in TBM in comparison to PTB (t test, *P*<0.05), and all are genes related to immune function. 5/11 genes were highly expressed in TBM in comparison to LTB (IL12B, PTGS2, MMP1, IL23A, and CCL20) however this did not reach statistical significance due to a consistent outlier in the LTB group (L2 which does not cluster with the other samples; see below). Twelve genes were highly expressed in PTB in comparison to LTB and TBM (PTB/LTB; MMP1, IL23A, HAS1, PTGS2, MGC10744, CCL20, CCL1, and IL12B, PTB/TBM; HAS1, KCNJ5, SERPINB7, and HS3ST2). 6/12 had significantly different expression levels (t test, *P*<0.05; Table 2). Nine genes were induced in LTB more than in other TB and 7 of these reached statistical significance (LTB/TBM; APOBEC3A, LTB/PTB P2RY13, MYO10, SLC39A8, CXCL11, F3, APOBEC3A, DUSP5). Together these results suggest that gene expression profiles in *Mtb*-stimulated macrophages may distinguish between the 3 different clinical forms of TB, LTB, PTB, and TBM.

**Table 2:** Thirty-three genes with altered expression ratios among different clinical forms of TB.

Gene Symbol	Characteristic	Means			Ratio	t-test^a^	ANOVA^b^
TBM/PTB		LTB	PTB	TBM	TBM/PTB	*P* value	p value
IL1B	immune cytokine	71.7	28.5	440.9	15.5	**0.013**	0.074
CXCL5	immune chemokine	3.0	1.4	18.6	13.7	**0.006**	**0.016**
EREG	immune signaling	70.5	25.0	331.5	13.3	**0.001**	**0.024**
TNIP3	immune signaling	39.6	10.5	131.9	12.5	**0.002**	**0.002**
IL1B	immune cytokine	51.7	26.5	285.2	10.8	**0.013**	0.095
CCR2	immune chemokine	0.2	0.1	0.7	10.6	**0.026**	0.133
INHBA	immune signaling	50.2	8.4	86.4	10.3	**<0.001**	**0.039**
**TBM/LTB**					**TBM/LTB**		
IL12B	immune cytokine	0.8	1.7	97.0	123.9	0.074	0.083
PTGS2	immune signaling	11.6	3131.3	1027.4	88.5	0.184	0.236
MMP1	extracellular matrix	0.1	16.3	3.8	59.4	0.247	0.115
IL23A	immune cytokine	0.2	1.2	9.2	42.9	0.184	0.110
CCL20	immune chemokine	22.1	319.4	393.8	17.9	0.824	0.952
**PTB/LTB**					**PTB/LTB**		
MMP1	extracellular matrix	0.1	16.3	3.8	256.7	0.088	0.115
IL23A	immune cytokine	0.2	16.8	9.2	78.5	0.099	0.110
HAS1	extracellular matrix	1.9	73.3	1.6	39.5	**0.007**	**0.001**
PTGS2	immune signaling	11.6	248.2	1027.4	21.4	0.559	0.236
MGC10744	hypothetical protein	1.4	23.4	2.3	16.3	**<0.001**	**<0.001**
CCL20	immune chemokine	22.1	319.4	393.8	14.5	0.830	0.952
CCL1	immune chemokine	1.5	18.8	3.2	12.8	**0.004**	**0.004**
IL12B	immune cytokine	0.8	9.8	97.0	12.5	0.481	0.083
**PTB/TBM**					**PTB/TBM**		
HAS1	extracellular matrix	1.9	73.3	1.6	47.2	**0.005**	**0.001**
KCNJ5	immune receptor	0.1	0.7	0.0	42.2	**<0.001**	**0.001**
SERPINB7	serine proteinase inhibitor	1.7	21.9	1.0	21.5	**0.025**	**0.016**
HS3ST2	transferase activity	0.5	1.0	0.1	11.1	**<0.001**	**0.005**
**LTB/TBM**					**LTB/TBM**		
APOBEC3A	hydrolase activity	33.3	2.8	1.5	22.0	**0.041**	**0.024**
HS3ST3B1	non immu signaling	26.8	6.4	2.2	12.0	0.090	0.100
**LTB/PTB**					**LTB/PTB**		
P2RY13	purinergic receptor	1.0	0.0	0.1	27.1	**0.018**	0.067
LOC348938	hypothetical protein	17.1	0.9	10.0	18.7	0.123	0.158
MYO10	myosin X	39.1	2.5	6.1	15.8	**0.003**	**0.002**
SLC39A8	solute carrier	43.7	3.0	16.7	14.6	**0.001**	**0.001**
CXCL11	immune chemokine	78.0	6.0	36.7	13.1	**0.003**	**0.002**
F3	coagulation factor	191.1	15.6	114.6	12.3	**0.011**	**0.011**
APOBEC3A	hydrolase activity	33.3	2.8	1.5	12.0	**0.024**	**0.024**
DUSP5	phosphatase	37.2	3.5	7.7	10.7	**0.005**	**0.016**

a t-test was used to compare means between the 2 indicated clinical groups.

b ANOVA was used to compare means among the 3 clinical groups.

*P* values<0.05 in bold.

### Validation results

We used real-time PCR using a TaqMan Low Density Array technique to confirm microarray results in 86 genes in an extended sample set which included 12 LTB, 12 PTB, and 10 TBM individuals. Fifty-eight of the 86 genes were selected from the microarray data based on high levels of induction (>15 fold) or repression (>5 fold) following *Mtb* stimulation. Forty six genes were selected based on array expression differences among the 3 clinical groups (>5 fold). We first assessed whether the expression patterns of the 58 up and down-regulated genes were replicated in the independent sample set using RT-PCR. In total, 90% (52/58) of the microarray results were confirmed by RT-PCR when assessing *Mtb* and PBS-stimulated expression values in the validation sample set (Table 3 and Table S2). The RT-PCR results showed that 5/58 genes (IFIT1, CXCL6, MERTK, CD36, and MS4A6A) were not significantly induced or repressed by *Mtb* stimulation (n = 34; *P*>0.05 by t-test) and the expression pattern of one gene, CCR2, was reversed (Table 3). In addition, the majority of the genes in the validation group (n = 34) had a higher induction level in comparison to the microarray group (n = 12; Table 3).

**Table 3:** Validation results of *Mtb* stimulated macrophage gene expression of 58 genes in 34 subjects.

Up-regulated genes	Gene	Microarrays	aFDR	cLDA	t-test	Gene description
Gene function		bratio	%	bratio	*P* value	
***Immunology***						
*Chemokines*	CCL20	29.7	0.0E+00	682.7	2.31E-19	C-C motif, ligand 20
	CXCL1	27.9	0.0E+00	181.4	1.39E-03	C-X-C motif, ligand 1
	CXCL11	21.6	0.0E+00	127.4	1.86E-11	C-X-C motif, ligand 11
	CXCL6	19.9	0.0E+00	670.8	**0.065**	C-X-C motif, ligand 6 (granulocyte chemotactic protein 2)
	CCL3	12.8	0.0E+00	26.8	3.23E-10	C-C motif, ligand 3
	GPR109B	9.6	0.0E+00	12.7	7.48E-07	chemokine receptor, G protein-coupled receptor 109B
	CXCL10	3.6	3.0E-01	24.1	8.83E-06	C-X-C motif, ligand 10
*Cytokines*	IL1A	101.3	0.0E+00	1468.2	5.05E-40	interleukin 1, alpha
	IL6	101.3	0.0E+00	853.1	1.55E-33	interleukin 6 (interferon, beta 2)
	IL1B	34.0	0.0E+00	688.6	2.78E-24	interleukin 1, beta
	CCL4	33.3	0.0E+00	1911.6	1.93E-09	C-C motif, ligand 4
	CXCL3	19.2	0.0E+00	156.6	5.74E-13	C-X-C motif, ligand 3
	IL10	12.3	0.0E+00	6.1	9.75E-04	interleukin 10
	IL1F9	12.0	0.0E+00	100.4	5.61E-10	interleukin 1 family, member 9
	CXCL2	11.5	0.0E+00	19.3	3.66E-08	C-X-C motif, ligand 2
	PBEF1	9.7	0.0E+00	15.4	1.20E-07	Pre-B-cell colony enhancing factor 1
	IL12B	6.5	0.0E+00	3350.8	2.78E-24	interleukin 12B
	CCL8	5.8	0.0E+00	33.8	9.59E-08	C-C motif, ligand 8
*Receptors*	CD80	12.6	0.0E+00	10.2	9.76E-07	CD80 antigen (CD28 antigen ligand 1, B7-1 antigen)
	TNFRSF4	6.0	0.0E+00	8.4	8.73E-08	tumor necrosis factor receptor superfamily, member 4
*Signaling*	PTX3	56.0	0.0E+00	182.2	5.06E-17	pentaxin-related gene, rapidly induced by IL-1 beta
	EREG	44.3	0.0E+00	61.7	1.83E-13	epidermal growth factor family
	PTGS2	26.8	0.0E+00	351.3	8.86E-16	prostaglandin-endoperoxide synthase 2
	TNFAIP6	24.2	0.0E+00	284.2	1.11E-17	tumor necrosis factor, alpha-induced protein 6
	IFIT1	22.2	0.0E+00	24.9	**0.903**	interferon-induced protein with tetratricopeptide repeats 1
	IRAK2	16.0	0.0E+00	6.7	1.54E-05	interleukin-1 receptor-associated kinase 2
	TNIP3	14.6	0.0E+00	11294.5	1.12E-14	TNFAIP3 interacting protein 3
	TRAF1	14.4	0.0E+00	11.5	1.34E-07	TNF receptor-associated factor 1
	INHBA	13.1	0.0E+00	88.9	4.19E-09	TGF-beta superfamily members
	IFIT2	10.3	0.0E+00	86.1	8.27E-04	interferon-induced protein with tetratricopeptide repeats 2
	IFIT3	9.0	0.0E+00	49.6	1.24E-09	interferon-induced protein with tetratricopeptide repeats 3
***Not immunology***						
*Receptors*	CD44	8.0	0.0E+00	2.6	1.43E-02	CD44 antigen
*Signaling*	JAG1	11.0	0.0E+00	13.1	3.02E-07	jagged 1 (Alagille syndrome)
	INSIG1	8.7	0.0E+00	19.5	1.03E-07	insulin induced gene 1
*Matrix*	PLAUR	9.0	0.0E+00	9.8	1.02E-06	plasminogen activator, urokinase receptor
	THBS1	6.5	0.0E+00	3.2	1.75E-02	thrombospondin 1
	MMP19	5.7	0.0E+00	13.6	8.35E-07	extracellular matrix
*Other*	SOD2	30.5	0.0E+00	7.5	1.81E-03	superoxide dismutase 2, mitochondrial
	F3	27.0	0.0E+00	7.0	3.58E-06	coagulation factor III (thromboplastin, tissue factor)
	SERPINB2	19.6	0.0E+00	57.5	4.78E-06	serine proteinase inhibitor, member 2
	G0S2	17.5	0.0E+00	81.1	1.63E-13	putative lymphocyte G0/G1 switch gene
	HEY1	17.3	0.0E+00	49.3	3.32E-10	hairy/enhancer-of-split related with YRPW motif 1
	PHLDA2	15.6	0.0E+00	12.3	1.17E-03	pleckstrin homology-like domain, family A, member 2
	SGPP2	14.3	0.0E+00	15.9	4.36E-09	Sphingosine-1-phosphate phosphotase 2
	OASL	12.7	0.0E+00	39.5	2.35E-12	2′-5′-oligoadenylate synthetase-like
	MET	9.0	0.0E+00	33.0	8.68E-08	met proto-oncogene (hepatocyte growth factor receptor)
	FNDC3B	8.2	0.0E+00	3.5	2.36E-03	fibronectin type III domain containing 3B
	IFI44L	6.0	0.0E+00	11.3	1.49E-04	interferon-induced protein 44-like
**Down-regulated genes**						
***Immunology***						
*Chemokines*	CCR2	**0.2**	4.1E+00	**6.2**	3.93E-04	C-C motif, receptor 2
*Signaling*	BIRC1	0.1	0.0E+00	0.2	1.1103E-18	baculoviral IAP repeat-containing 1
	GLUL	0.2	4.4E-01	0.3	2.50E-02	glutamate-ammonia ligase (glutamine synthase)
	MERTK	0.2	9.1E-02	0.4	**0.090**	c-mer proto-oncogene tyrosine kinase
*Receptor*	KCNJ5	0.1	5.9E-01	0.1	1.04E-03	Potassium inwardly-rectifying channel, member 5
*Other*	P2RY13	0.2	1.3E+00	0.3	7.67E-04	purinergic receptor P2Y, G-protein coupled, 13
	DAB2	0.2	1.1E+00	0.2	3.41E-03	disabled homolog 2, mitogen-responsive phosphoprotein
	CD36	0.3	3.0E+00	0.6	**0.505**	CD36 antigen (collagen type I receptor)
	MS4A6A	0.3	0.0E+00	0.6	**0.551**	membrane-spanning 4-domains, subfamily A, member 6A
	STAC	0.5	8.4E+00	0.5	1.44E-02	SH3 and cysteine rich domain

a FDR = false discovery rate of microarrays using SAM.

b ratio indicates the mean of *Mtb* stimulated samples divided by the mean of PBS stimulated samples with data derived from microarray (n = 12) or.

c LDA real-time PCR (n = 34).

**Figure 1:**
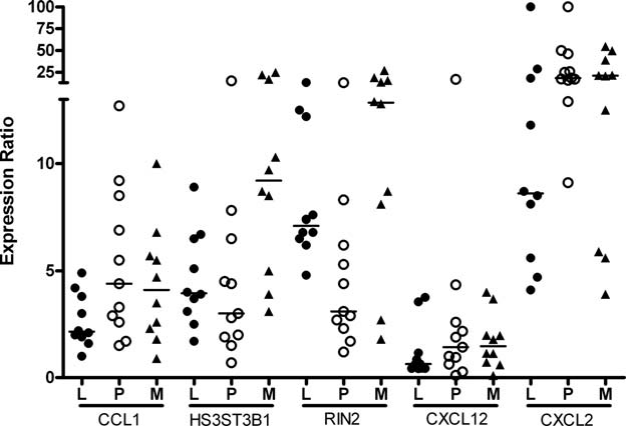
Scatter plots of gene expression from 3 TB clinical groups. mRNA expression values from the RT-PCR validation step are depicted from 5 representative genes (n = 34). Expression values are ratios of *Mtb*-stimulated gene expression in comparison to PBS-stimulation in LTB (dark round), PTB (empty round), and TBM (triangle).

We next compared gene expression levels in the 3 clinical groups in the validation sample set. The RT-PCR results showed that 2/46 genes (CCL1 and HS3ST3B1) were differentially expressed in groups with different TB phenotype (*P*<0.05 by t-test; Table 4). CCL1 was up-regulated in PTB when compared to LTB in both the RT-PCR LDA validation samples (*P* = 0.02 by t-test; 1.9-fold) and the initial microarray analysis (12.8-fold; Table 4 and Table S3). HS3ST3B1 was down regulated in LTB when compared to TBM in the RT-PCR LDA validation samples (*P* = 0.008 by t-test; ratio = 0.4) but this pattern of expression was reversed in the initial microarray analysis (ratio = 12.8) (Table 4). Scatter plots of CCL1 and HS3ST3B1 are shown in Figure 1 along with 3 other representative genes. Seven other genes (INHBA, TSLP, LY6K, IL12B, MMP1, CCL20 and HAS1) had a greater than 2-fold change in expression ratios of the validation samples in each pair-wise comparison, but these differences did not reach statistical significance (*P*>0.05; Table 4). These results suggest that the different TB clinical phenotypes cannot easily be distinguished by examining expression levels of single genes.

**Table 4:** Validation results for 46 genes with altered expression ratios among different clinical forms of TB.

	aComparison	aComparison		Gene function
	Microarray	LDA	t-test	
Gene name	TBM/PTB	TBM/PTB	*P* value	
INHBA	15.5	2.8	0.347	immune signaling
IL1B	15.5	1.0	0.901	immune cytokine
TNIP3	12.5	0.1	0.354	immune signaling
CCR2	10.6	1.2	0.596	immune chemokine
CTHRC1	9.5	0.6	0.585	extracellular matrix
STAC	7.9	1.8	0.254	metal ion binding
TSLP	6.8	2.8	0.266	immune cytokine
SLC16A10	6.5	0.8	0.322	menbrane transporter
CXCL11	6.2	1.8	0.369	immune chemokine
CHIT1	5.8	0.8	0.342	chitotriosidase
LY6K	5.8	6.4	0.269	immune receptor
PBEF1	5.5	1.4	0.413	immunity and defense
CXCL12	6.2	0.6	0.354	immune chemokine
UBE3A	8.0	1.1	0.660	ligase activity
CD69	8.2	1.1	0.737	immune receptor
SF1	8.0	1.6	0.219	RNA splicing
CNR1	7.2	0.5	0.128	neuroactive ligand-receptor
GSTA4	6.2	1.3	0.297	cell growth factor
MT1H	5.9	0.9	0.763	metal ion binding
SLITRK6	5.7	0.0	0.324	protein binding
RGS1	5.7	1.3	0.509	immune signaling
TAOK1	5.3	1.0	0.943	kinase
COCH	7.8	0.7	0.116	coagulation factor
RASGEF1B	8.8	1.4	0.258	guanyl-nucleotide exchange factor
CD36	9.5	1.2	0.446	immune receptor
BCL2L14	6.6	1.1	0.833	protein binding in regulation of apoptosis
GRK5	5.2	1.0	0.811	non immune signaling
	**TBM/LTB**	**TBM/LTB**		
IL12B	123.9	3.4	0.511	immune cytokine
MMP1	59.4	3.1	0.775	extracellular matrix
IL23A	42.9	1.8	0.381	immune cytokine
CCL20	17.9	2.5	0.065	immune chemokine, MIP3A
	**PTB/LTB**	**PTB/LTB**		
HAS1	39.5	3.7	0.407	extracellular matrix
CCL1	12.8	1.9	**0.020**	immune chemokine
	**PTB/TBM**	**PTB/TBM**		
HS3ST2	11.1	3.0	0.095	transferase activity
	**LTB/TBM**	**LTB/TBM**		
HS3ST3B1	12.0	0.4	**0.008**	non immune signaling
APOBEC3A	22.0	0.4	0.277	hydrolase activity
PSD3	6.1	0.9	0.781	guanyl-nucleotide exchange factor
FCAR	6.4	0.6	0.178	immune receptor
RIN2	6.5	0.7	0.149	guanyl-nucleotide exchange factor
	**LTB/PTB**	**LTB/PTB**		
P2RY13	27.1	0.5	0.375	non immune signaling
MMP19	5.5	1.8	0.157	extracellular matrix
AK3	5.7	0.6	0.099	kinase
CXCL10	6.6	1.3	0.434	immune chemokine
HLA-DOB	7.3	0.8	0.163	immune receptor
CD24	8.1	1.2	0.607	immune receptor
CSF2	9.5	0.2	0.356	immune cytokine

a The comparison was calculated from ratios of mean values of the *Mtb*-stimulated samples over PBS-stimulated samples in each clinical group (LTB n = 12, PTB n = 12, and TBM n = 10). t-test was used to compare means between the 2 indicated clinical groups from the LDA data.

### Cluster analysis of the 3 clinical phenotypes

We next hypothesized that expression profiles from multiple genes would need to be combined to detect patterns that could distinguish the different clinical disease phenotypes. We selected 1,608 highly induced or repressed genes from the microarray data set (Table S1) and used an unsupervised, hierarchical clustering algorithm [21] of 12 individual samples to attempt to distinguish the profiles of the 3 groups (Figure S1). These results show that, (1) there was more relatedness between expression levels of samples from the same clinical group, i.e. L1 and L3 are very similar, P1, P2 and P3 are very similar, and M1 and M4 are very similar, and (2) one large cluster containing data from all TBM subjects, all PTB subjects and one LTB subject (L4) is very distinct to data from subjects L2, L1 and L3. Together, these findings suggest that cluster analysis can partially distinguish different clinical forms of TB.

### CCL1 SNPs are associated with TB

**Figure 2:**
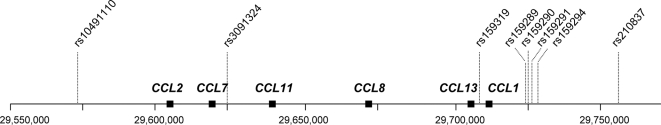
The CCL gene cluster containing *CCL1* on chromosome 17. The black boxes denote the genes that are found in this region and the TB associated SNPs are approximately located by the dashed lines. *denotes gene encoding a hypothetical protein. Not to scale.

CCL1 was the only gene whose expression was up-regulated in both the microarray and validation data sets when comparing clinical forms of TB (PTB vs LTB). We next examined whether genetic variants of *CCL1* were associated with susceptibility to TB in a case-control study with TBM (N = 114) and PTB patients (N = 159), and population controls (N = 188) by using gene chip mapping assays. Forty nine SNPs were genotyped across a 200 kb region of the chromosome 17 CCL gene family cluster. Eight of the forty nine SNPs were associated with TB. To further locate the region associated with TB, we arbitrarily divided the whole region into four 50 kb sections. The first section containing CCL2 had 1/9 associated SNPs, the second containing CCL7 and CCL11 had 1/9 associated SNPs, the third containing CCL8 and CCL13 had 1/7 associated SNPs and the fourth containing CCL1 had 4/23 associated SNPs (Figure 2). To investigate this further we genotyped 10 SNPs nearby and in the coding region of CCL1 using Sequenom. Two more SNPs in the CCL1 gene were significantly associated with TB by genotypic comparison (Table 5). Together these results suggest that polymorphisms near and within the CCL1 genomic region are associated with susceptibility to different TB phenotypes.

**Table 5:** CCL1 SNP allele and genotype frequencies in control and TB groups.

SNP, group	Position			Allele		Genotype			Allelic comparison		Genotypic comparison
		1	2	1	2	11	12	22	OR^a^ (95%CI^b^)	*P* value	*P* value
**rs10491110**	29572630	T	C								
Control				296 (0.79)	80 (0.21)	119 (0.63)	58 (0.31)	11 (0.06)			
cAll TB				554 (0.84)	104 (0.16)	233 (0.71)	88 (0.27)	8 (0.02)	0.7 (0.5–0.9)	d **0.027**	0.063
PTB				297 (0.83)	63 (0.18)	122 (0.68)	53 (0.29)	5 (0.03)	0.9 (0.5–1.1)	0.196	0.311
TBM				257 (0.86)	41 (0.14)	111 (0.75)	35 (0.24)	3 (0.02)	0.6 (0.4–0.9)	**0.012**	**0.047**
TBM/PTB									0.7 (0.5–1.1)	0.190	0.408
**rs3091324**	29625029	C	A								
Control				270 (0.73)	98 (0.27)	101 (0.55)	68 (0.37)	15 (0.08)			
All TB				478 (0.73)	178 (0.27)	174 (0.53)	130 (0.40)	24 (0.07)	1.0 (0.7–1.3)	0.862	0.818
PTB				276 (0.77)	84 (0.23)	104 (0.58)	68 (0.38)	8 (0.04)	0.8 (0.6–1.1)	0.304	0.344
TBM				202 (0.68)	94 (0.32)	70 (0.47)	62 (0.42)	16 (0.11)	1.2 (0.9–1.7)	0.147	0.367
*TBM/PTB									**1.5 (1.0–2.1)**	**0.016**	**0.038**
**rs2072069**	29709104	A	G								
Control				375 (0.50)	373 (0.50)	91 (0.24)	193 (0.52)	90 (0.24)			
All TB				318 (0.48)	338 (0.52)	88 (0.27)	142 (0.43)	98 (0.30)	1.1 (0.9–1.3)	0.535	0.098
PTB				163 (0.47)	183 (0.53)	40 (0.23)	83 (0.48)	50 (0.29)	1.1 (0.9–1.5)	0.352	0.481
TBM				155 (0.50)	155 (0.50)	48 (0.31)	59 (0.38)	48 (0.31)	1.0 (0.8–1.3)	0.968	**0.015**
*TBM/PTB									0.9 (0.7–1.2)	0.459	0.175
**rs159319**	29710800	A	G								
Control				210 (0.56)	166 (0.44)	56 (0.30)	98 (0.52)	34 (0.18)			
All TB				332 (0.51)	322 (0.49)	83 (0.30)	166 (0.51)	78 (0.24)	1.2 (1.0–1.6)	0.115	0.256
PTB				170 (0.48)	188 (0.53)	39 (0.22)	92 (0.51)	48 (0.27)	1.4 (1.0–1.8)	**0.023**	0.067
TBM				162 (0.55)	134 (0.45)	44 (0.30)	74 (0.50)	30 (0.20)	1.0 (0.7–1.4)	0.771	0.869
*TBM/PTB									0.7 (0.5–1.0)	0.065	0.173
**rs3138031**	29712619	A	C								
Control	intron CCL1			684 (0.95)	38 (0.05)	324 (0.90)	36 (0.10)	1 (0.00)			
All TB				481 (0.94)	29 (0.06)	230 (0.90)	21 (0.08)	4 (0.02)	1.1 (0.7–1.8)	0.747	0.174
PTB				240 (0.92)	22 (0.08)	113 (0.86)	14 (0.11)	4 (0.03)	1.7 (1.0–2.8)	0.069	**0.024**
TBM				241 (0.97)	7 (0.03)	117 (0.94)	7 (0.06)	0 (0.00)	0.5 (0.2–1.2)	0.114	0.145
*TBM/PTB									0.3 (0.1–0.7)	0.006	0.064
**rs159290**	29725037	T	C								
Control				212 (0.56)	164 (0.44)	58 (0.31)	96 (0.51)	34 (0.18)			
All TB				340 (0.52)	316 (0.48)	85 (0.26)	170 (0.52)	73 (0.22)	1.2 (0.9–1.5)	0.158	0.355
PTB				171 (0.48)	187 (0.52)	38 (0.21)	95 (0.53)	46 (0.26)	1.4 (1.1–1.9)	**0.019**	0.056
TBM				169 (0.57)	129 (0.43)	47 (0.32)	75 (0.50)	27 (0.18)	1.0 (0.7–1.4)	0.933	0.989
*TBM/PTB									1.4 (1.1–1.8)	**0.022**	0.062
**rs159291**	29725240	C	T								
Control				207 (0.56)	165 (0.44)	55 (0.30)	97 (0.52)	34 (0.18)			
All TB				339 (0.52)	319 (0.48)	84 (0.26)	171 (0.52)	74 (0.23)	1.2 (0.9–1.5)	0.202	0.422
PTB				170 (0.47)	190 (0.53)	37 (0.21)	96 (0.53)	47 (0.26)	1.4 (1.1–1.8)	**0.023**	0.063
TBM				169 (0.57)	129 (0.43)	47 (0.32)	75 (0.50)	27 (0.18)	1.0 (0.7–1.3)	0.782	0.923
*TBM/PTB									0.7 (0.5–0.9)	**0.015**	0.052
**rs159294**	29728905	T	A								
Control				333 (0.89)	43 (0.11)	145 (0.77)	43 (0.23)	0 (0.00)			
All TB				534 (0.82)	122 (0.19)	217 (0.66)	100 (0.31)	11 (0.03)	1.8 (1.2–2.6)	**0.003**	**0.004**
PTB				286 (0.79)	74 (0.21)	114 (0.63)	58 (0.32)	8 (0.04)	2.0 (1.3–3.0)	**<0.001**	**0.001**
TBM				248 (0.84)	48 (0.16)	103 (0.70)	42 (0.28)	3 (0.02)	1.5 (1.0–2.3)	0.070	0.065
*TBM/PTB									0.7 (0.5–1.1)	0.155	0.318
**rs210837**	29759282	C	T								
Control				331 (0.88)	45 (0.12)	143 (0.76)	45 (0.24)	0 (0.00)			
All TB				542 (0.83)	114 (0.17)	219 (0.67)	104 (0.32)	5 (0.02)	1.5 (1.1–2.2)	**0.021**	**0.032**
PTB				292 (0.82)	66 (0.18)	117 (0.65)	58 (0.32)	4 (0.02)	1.6 (1.1–2.5)	**0.014**	**0.018**
TBM				250 (0.84)	48 (0.16)	102 (0.69)	46 (0.31)	1 (0.01)	1.4 (0.9–2.2)	0.122	0.182
*TBM/PTB									0.8 (0.6–1.3)	0.434	0.477

a For odds ratio (OR) calculation each group was compared with the control group, except for OR calculation for TBM/PTB, where TBM was compared with PTB.

b CI, confidence inte.

c All TB represents the combination of PTB and TBM.

d numbers in bold represent P values<0.05.

### Regulation of CCL1 Expression

**Figure 3:**
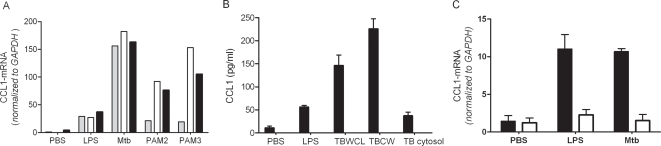
The cellular function of CCL1. (A) CCL1 mRNA expression in cells stimulated with *Mtb* and TLR ligands. Real-time PCR quantification of CCL1 in THP1 (grey columns), U937 (open columns), and PBMC (solid columns). Cells were stimulated with LPS (100 ng/ml), whole cell H37Rv *Mtb* lysates (50 µg/ml), and lipopeptides PAM2 or PAM3 (250 ng/ml). Cells were stimulated for 4 hours and mRNA was extracted and measured by real-time PCR. (B) CCL1 secretion in PBMCs stimulated with *Mtb*. PBMCs were stimulated with LPS (100 ng/ml), whole cell H37Rv *Mtb* lysates (50 µg/ml), TB cell wall (5 µg/ml), or TB cytosol (5 µg/ml). After stimulation for 24 hours, supernatants were assayed for CCL1 production by ELISA. Values represent mean and standard deviation of triplicate samples. Student's t-test for comparisons between non-stimulated and either LPS, TBWCL, TBCW, or TB cytosol have *P* = 0.002, 0.01, 0.006, and 0.02, respectively. (C) CCL1 expression in BMM from *Myd88−/−* (open columns) and wild-type mice (solid columns). BMM were stimulated with LPS (100 ng/ml) or *Mtb* (100 µg/ml) for 4 hours. Means of CCL1 expression examined in triplicate by real-time PCR are shown.

To further investigate the role of CCL1 in *Mtb* pathogenesis, we examined regulation of its expression. We found that CCL1 mRNA expression was cell-specific and highly induced in monocytic (THP-1, U937, & PBMCs) cells stimulated with *Mtb* lysates or TLR ligands (LPS, PAM2, PAM3) (Figure 3A). In contrast, no expression was found in epithelial cell lines (HeLa & A549, data not shown). We also found that CCL1 protein secretion was induced in THP1 cells and PBMCs by *Mtb*, including whole cell lysates, cell wall and cytosolic fractions [Figure 3B and data not shown; PBS vs TB whole cell lysate (TBWCL; P = 0.01), PBS vs TB cell wall (TBCW; P = 0.006) and PBS vs TB cytosol (P = 0.02)]. Finally, we examined CCL1 expression in murine bone-marrow derived macrophages stimulated with PBS, LPS or *Mtb* from wild-type (WT) and *Myd88−/−* mice. CCL1 expression was highly induced by LPS and *Mtb* in WT bone marrow macrophages (BMMs). However, CCL1 expression was decreased in MyD88-deficient BMMs stimulated with LPS (P = 0.03) or *Mtb* (P = 0.002) (Figure 3C). Together, these results suggested that CCL1 expression is highly enriched in monocytes and induced by *Mtb* components in a MyD88-dependent manner.

## Discussion

In this study we examined macrophage transcriptional profiles in individuals with different clinical forms of TB. The majority of reported TB microarray studies have examined healthy donors, cell lines or murine cells [13]–[17]. Only one previous study has compared gene expression profiles of individuals with different clinical forms of TB [24]. Mistry *et al* obtained whole blood from individuals with active, latent, cured (following 1 disease episode) and recurrent TB (following 2–3 episodes) [24]. Discriminant analysis suggested that 9 genes could distinguish the 4 clinical TB groups [24]. We examined these 9 genes in our data set and found these genes could not differentiate our latent and cured TB groups. These differences may be attributable to the study design, which was substantially different from the current investigation with regard to cell population (whole blood vs MDMs), stimuli (none vs whole cell *Mtb* lysate), ethnic background (South African vs Vietnamese) and comparison of different clinical phenotypes. Despite these methodologic differences, both studies suggest that host gene expression profiles uniquely identify groups of individuals with different types of TB. Our study further illustrates that macrophages, the primary host cell involved in TB pathogenesis, are a key source of the unique transcriptional profile that distinguishes clinical forms of TB.

One limitation of our study was the small sample size. Although this is the largest number of individuals ever studied in a TB microarray study, comparable only to the study by Mistry *et al* [24], the sample size remains small for this statistically challenging question. To overcome some of the limitations of a small sample size for microarrays (n = 12), we included an independent set of samples for validation (n = 34). We also chose to use a whole cell lysate of a standardized *Mtb* strain rather than live organisms and a relatively short stimulation time (t = 4 hours) to minimize variation in our stimulation conditions and to enhance the detection of early innate immune response genes. We examined these cells in an *ex vivo* environment to avoid variability that is attributable to complex *in vivo* environments. For example, we studied individuals after they had been treated for TB to avoid detecting gene expression changes that are attributable to stimulation of *in vivo* inflammatory pathways from active disease. We also chose to study macrophages rather than whole blood in order to concentrate on a single cell population that is most relevant for Tb pathogenesis. A number of studies have shown that the strain of *Mtb* induces different immune responses [25],[26]. Although the choice of *Mtb* strain could stimulate different gene expression profiles, we chose to study the commonly used laboratory strain (*Mtb* H37Rv). Each of these experimental conditions was selected to maximize the opportunities of detecting differences attributable to genetic variation in the macrophage innate immune response to TB. Comparison of gene expression results with alternative experimental conditions (such as different cell types, *Mtb* strains, *Mtb* growth conditions, and time points) could further illuminate the role of these genes in Tb pathogenesis.

In addition to comparing expression profiles among people with different types of TB, our study contributes further data on the set of genes that are activated in response to *Mtb* stimulation of macrophages. Our results demonstrated that 1,608 genes in macrophages were stimulated (up or down-regulated) by *Mtb*. Furthermore, 90% of a subset of these genes (n = 58 genes induced >15 fold by *Mtb* stimulation) in a second round validation also showed altered expression. Many genes identified in our study have also been detected in previous studies investigating the host response to *Mtb* infection [13],[16],[17]. Ragno *et al* studied THP-1 cells stimulated with live TB and measured the expression of 375 genes after 6 or 12 hours of stimulation. Our data confirmed 15 genes significantly induced following 6 hr stimulation in their data set (MIP-1α, MIP-1β, MIP-3α, MPIF-1, PARC, RANTES, IL-8, GRO-α, GRP-β, GRO-γ, CCL1, CCR3, IL-1β, TNFα, and VEGF) [17]. Nau *et al* studied primary human MDMs stimulated with live *Mtb* [16]. Eleven genes were highly expressed in both data sets (TNFAIP6, CXCL3, CXCL1, CCL4, PTGS2, SERPINB2, PTX3, INHBA, TRAF1, JAG1, and SOD2) and 3 genes were inhibited (MERTK, GLUL, and DAB2). These gene lists include cytokines, chemokines and immune receptors, which may be involved in inflammatory responses in the early phases of defense against *Mtb*. All of the up-regulated genes identified by Nau *et al* were found in our dataset [16]. In contrast, only 50% (24/50) of highly expressed genes in our dataset were identified by Nau *et al*, a difference that is likely due to the array sizes that were utilized (38,000 vs. 980 genes). Although these microarray studies have important methodologic differences (e.g primary cells vs cell lines, healthy subjects vs. TB patients, live versus dead *Mtb* stimulation, stimulation times, arrays and genes analyzed), all of these studies have identified novel genes potentially related to the host macrophage response to *Mtb*.

Our study compares transcriptional profiles of individuals with TBM with individuals with other forms of TB. We identified genes that were distinctly expressed in macrophages from individuals with a history of TBM. After bacilli invade the host lung within the pulmonary alveolar macrophage, they replicate and disseminate to the regional lymph nodes. During this early stage of infection, before the development of adaptive immunity, the bacteria can spread haematogenously to other organs in the body and cause extrapulmonary disease, such as TBM [27],[28]. This step may be determined by the nature and extent of the innate immune response activated by infected macrophages. We found that several macrophage immune response genes (IL1B, IL12B, TNF, TNIP3, CXCL10, CXCL11, CCL12, and CCL1) were up-regulated in TBM subjects in comparison to those with PTB and LTB. In addition, some genes, such as MMP1 and HAS1, were found with differing expression in PTB and TBM patients. These genes are involved in degrading the extracellular matrix and could mediate a role in granuloma formation and bacillus containment, which could influence dissemination and development of TBM [23]. Although the relationship between the inflammatory response and TBM pathogenesis is only partially understood, excessive immune activation may be intimately associated with disease severity and outcome.

Case-control genetic association studies of biologically plausible candidate genes have been performed with the hope to identify genes involved in susceptibility to, and clinical outcome of, TB. However it has always been challenging to identify potential candidate genes in an unbiased manner. The expression profiling study we describe here can serve as a hypothesis generating, unbiased methodological approach to identify genes for potential association studies. Despite this advantage, gene regulation is not the only mechanism for genetic resistance or susceptibility and non-synonymous coding region SNPs which alter protein structure and function also play an important role. From the genes that were differentially expressed between TB disease types, as assessed by microarray, we tested 46 genes in a separate, larger sample set by RT-PCR. The expression of only one of these genes, CCL1, remained significantly different between patients with different clinical TB outcomes. To test our selection approach we performed a case-control genetic association study and found that SNPs near CCL1 were associated with susceptibility to PTB. The fact that SNPs near CCL1 were significantly associated with PTB in our study highlights the feasibility of this unbiased selection approach.

Even though the associated SNPs are not within the CCL1 coding region, it is a likely candidate gene due to it's proximity to the cluster of associated SNPs and its functional relevance. CCL1, like other members of the CC chemokine family, is an inflammatory mediator that stimulates the migration of human monocytes [29]. CCL1 is produced by monocytes (as well as other cells) and binds its receptor CCR8, which is present on lymphocytes and monocytes [30]. Interestingly, CCR8 has enriched expression on Th2 and regulatory T cells and may influence the development of Th2 type T cell responses *in vivo* [31],[32]. In addition, CCR8 regulates migration of dendritic cells to lymph nodes [33]. Hoshino *et al* [34] found that the expression of CCR8 was specifically up-regulated by CCL1 stimulation of peritoneal macrophages, which may lead to cell aggregation at a site of tissue damage. In the lungs, CCL1 expression was up-regulated in *Mycobacterium bovis* purified protein derivative (PPD) induced granulomas [35]. In this study, we found that CCL1 expression was induced by *Mtb* and TLR ligands in several monocyte/macrophage lineages. Furthermore, we found that its expression was MyD88-dependent when cells were stimulated with LPS or *Mtb*. Genetic variation leading to the loss or alteration of CCL1 function may influence the ability of T cells, monocytes and dendritic cells to migrate to the site of infection, aggregate into granulomas and develop an effective immune response. This may result in inadequate containment of the bacterium and allow unimpeded bacterial growth leading to pulmonary disease.

With currently available tools, clinicians are unable to identify the subset of latently infected patients who will develop active disease. Furthermore, there are no techniques available to prospectively identify individuals at risk for the devastating consequences of TBM versus more treatable forms of TB such as localized pulmonary disease. Further studies in this area could lead to tests that could alter treatment algorithms with more accurate prognostic information. In addition, such studies may lead to novel molecular insight into TB pathogenesis.

## Supporting Information

Figure S1 Unsupervised hierarchical clustering analysis of 1,608 genes that are up or down regulated with a fold change of >2 in (a) 12 individual samples from LTB, PTB and TBM subjects. (b) A magnification of the dendogram from section a.
(0.95 MB DOC)

Table S1 1608 genes induced by *M.tb*
(0.24 MB XLS)

Table S2 Validation results of *M.tb*-stimulated MDM gene expression
(0.04 MB DOC)

Table S3 Validation results for genes with altered expression ratios among different clinical forms of TB
(0.04 MB DOC)
