# Supplementary material for: Identification of Tuberculosis Susceptibility Genes with Human Macrophage Gene Expression Profiles
Source: PLoS Pathog. 2008 Dec 5;4(12):e1000229. doi: 10.1371/journal.ppat.1000229 (PMC2585058; doi:10.1371/journal.ppat.1000229)
Supplement: Figure S1 — Unsupervised hierarchical clustering analysis of 1,608 genes that are up or down regulated with a fold change of >2 in (a) 12 individual samples from LTB, PTB and TBM subjects. (b) A magnification of the dendogram from section a. (0.95 MB DOC) [file ppat.1000229.s001.doc]

**Figure S1** Unsupervised hierarchical clustering analysis of 1,608 genes that are up or down regulated with a fold change of >2 in (a) 12 individual samples from LTB, PTB and TBM subjects. (b) A magnification of the dendogram from section a.


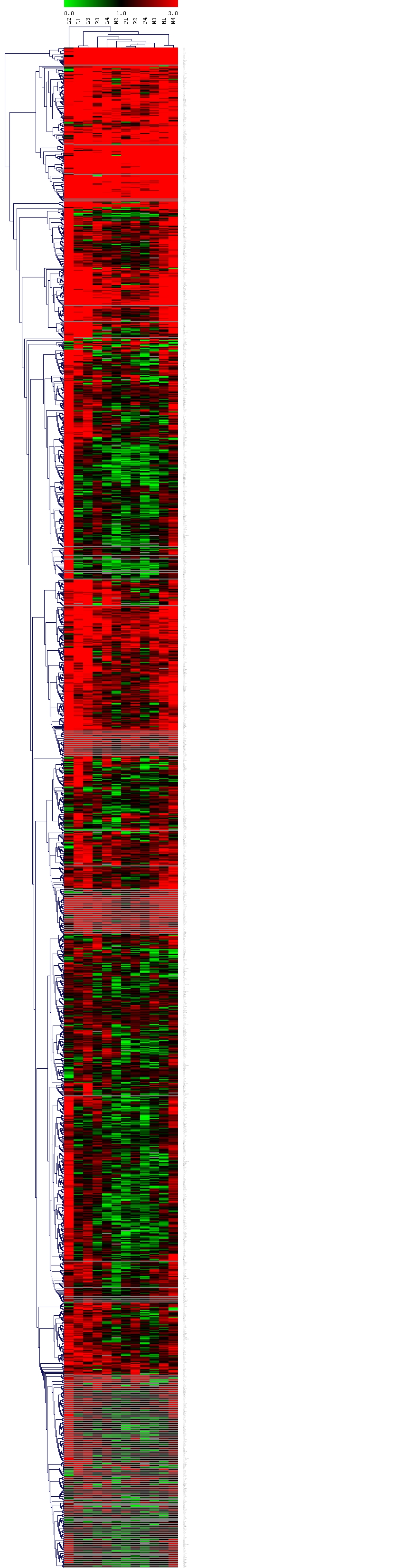

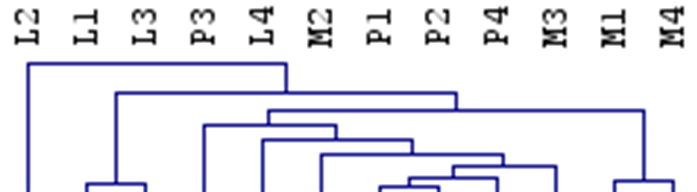
 (a) (b)
