# Supplementary material for: Identification of Tuberculosis Susceptibility Genes with Human Macrophage Gene Expression Profiles
Source: PLoS Pathog. 2008 Dec 5;4(12):e1000229. doi: 10.1371/journal.ppat.1000229 (PMC2585058; doi:10.1371/journal.ppat.1000229)
Supplement: Table S2 — Validation results of M.tb-stimulated MDM gene expression (0.04 MB DOC) [file ppat.1000229.s003.doc]

**Table S2** Validation results of *M.tb*-stimulated MDM gene expression

**Supplementary Table 2** (continued)

Note. t-test was used to compare means between stimulated and un-stimulated groups. Mean values, standard deviations and ratios of gene expression in stimulated and un-stimulated MDMs from microarray and LDA experiments are presented. Genes in bold are not confirmed.
