# Supplementary material for: Identification of Tuberculosis Susceptibility Genes with Human Macrophage Gene Expression Profiles
Source: PLoS Pathog. 2008 Dec 5;4(12):e1000229. doi: 10.1371/journal.ppat.1000229 (PMC2585058; doi:10.1371/journal.ppat.1000229)
Supplement: Table S3 — Validation results for genes with altered expression ratios among different clinical forms of TB (0.04 MB DOC) [file ppat.1000229.s004.doc]

Table S3 Validation results for genes with altered expression ratios among different clinical forms of TB

Supplementary Table 3 (continued)

Note. Means are mean values of the M.tb stimulated samples over PBS stimulated samples in each clinical group (LTB n=12, PTB n=12 and TBM n=10). Std, standard deviation. T-test was used to compare means between the 2 indicated clinical groups from the LDA data.
